# Clinical significance of circulating tumour cells and tumour marker detection in the chemotherapeutic evaluation of advanced colorectal cancer

**DOI:** 10.1111/codi.15939

**Published:** 2021-10-23

**Authors:** Feifei Shen, Yiwen Zhu, Fan Wang, Xun Cai, Honghua Ding, Fei Zhou, Jingjue Wang, Hongli Gu, Chuan Liu, Qi Li

**Affiliations:** ^1^ Department of Oncology Shanghai General Hospital Shanghai Jiao Tong University School of Medicine Shanghai China

**Keywords:** colorectal cancer, CTCs, tumour markers

## Abstract

**Aim:**

Systemic chemotherapy combining biological targeted therapies is the standard therapy for patients with metastatic colorectal cancer (mCRC), but effective markers are needed to identify clinical responders. Circulating tumour cells (CTCs) have been associated with prognosis in patients with mCRC. This study aimed to explore the relationship between CTC number and the clinical response of patients with advanced CRC.

**Method:**

Epithelial cell adhesion molecule‐independent enrichment and CD45^−^ fluorescence *in situ* hybridization immunofluorescence were used to detect peripheral blood CTCs in 79 patients with advanced CRC. Fisher's exact test and Spearman's rank correlation coefficient were used to analyse the correlation between CTC number and efficacy of chemotherapy. Kaplan–Meier and Cox regression analyses were used to evaluate progression‐free survival (PFS).

**Results:**

Among the evaluable patients, CTCs were significantly correlated with clinical response (*r* =4.891, *p* = 0.031). High CTC numbers were associated with a poor treatment response (*r* = −0.250, *p* = 0.027). Dynamic decrease in CTC number was associated with clinical response (*p* = 0.046). High baseline CTC number and carcinoembryonic antigen levels were prognostic factors for unfavourable PFS in multivariable analysis [hazard ratio (HR) = 3.30, *p* = 0.011 and HR = 2.04, *p* = 0.044, respectively]. Compared with the CTC‐positive group, the CTC‐negative group showed superior PFS (median PFS 15.53 vs. 9.43 months, *p* = 0.041) among CRC patients receiving first‐line treatment.

**Conclusion:**

CTC number is a feasible biomarker for predicting outcomes in mCRC patients receiving systemic chemotherapy.

**What does this paper add to the literature?**

Circulating tumour cells have been associated with prognosis in patients with metastatic colorectal cancer (mCRC). CTC positivity at baseline, as well as without a reduction in the number of CTCs during chemotherapy, in mCRC patients is positively correlated with disease progression. CTC number may be used as a new biomarker for predicting disease progression and survival.

## INTRODUCTION

Colorectal cancer (CRC) is the third most common cancer and second most common cause of cancer‐related death worldwide. More than 1.9 million new cases of CRC and 935,000 deaths occurred worldwide in 2020 [1]. The incidence and mortality of CRC in China are increasing, with 383,000 new cases and 187,000 deaths in 2015 [2]. The incidence and mortality rates in China in 2018 were 23.7 per 100,000 and 10.9 per 100,000, respectively [3]. Metastasis is the main cause of death from CRC, and is present in approximately 25% of patients with CRC at initial diagnosis [4].

Systemic chemotherapy combining biological targeting therapies is the standard therapy for patients with metastatic colorectal cancer (mCRC). Although the use of these combination therapies in mCRC has led to response rates of >50%, approximately 28%–44% of patients have no response to first‐line treatment with double chemotherapy plus either cetuximab or bevacizuamb [5]. Assessment of tumour response by imaging and serum tumour markers has been associated with survival, but these measurements are not sufficient to predict the efficacy of systemic chemotherapy. Therefore, there is an urgent need to develop robust prognostic biomarkers for mCRC patients receiving chemotherapy to predict resistance and identify alternative strategies for overcoming chemotherapeutic resistance. Unfortunately, no accepted biological or molecular marker of prognostic value is currently available for systemic chemotherapy.

Circulating tumour cells (CTCs) are cancer cells that detach from the primary tumour or its metastases and disseminate in the bloodstream; these cells can be isolated directly from peripheral blood, obviating the need for invasive tumour biopsies [6, 7. Recent refinement of an immunomagnetic separation technology to reliably and reproducibly isolate, enumerate and characterize CTCs in epithelial malignancies has enabled further study of CTCs as a prognostic and predictive marker [8]. In recent years, CTCs have been widely proposed to serve as biomarkers in various cancer types, including breast, prostate and colorectal cancer [9, 10, 11, 12. Studies have also shown that CTC number is an independent predictor of progression‐free survival (PFS) and overall survival (OS) in mCRC [11, 12. Therefore, we hypothesized that CTC number might be a potential indicator for predicting the response to chemotherapy in patients with mCRC.

In this study, we aimed to investigate the impact of CTC number on the outcomes and prognoses of first‐line treatment in mCRC patients.

## METHOD

### Study design and patients

This retrospective observational single‐centre study included 79 patients with mCRC who had undergone first‐line treatment at Shanghai General Hospital between March 2016 and December 2020. Eligible patients were those aged 18–75 years who presented with Stage IV histologically confirmed adenocarcinoma of the colon or rectum; had an Eastern Cooperative Oncology Group (ECOG) performance status of 0–2; and had never received radiotherapy, chemotherapy, targeted therapy or other forms of treatment after metastatic disease was diagnosed. Patients were excluded if they had second malignancies or multiple primary malignancies. All the included patients were scheduled to undergo first‐line treatment (chemotherapy with or without biological targeting therapies). The study was approved by the Ethics Board of Shanghai General Hospital. Informed consent to participate in the study was obtained from all the patients.

### Collection, enrichment and identification of circulating tumour cells

Peripheral blood samples were collected by venepuncture. For CTC tests, 3.2 ml of blood was used after discarding the first 1.8 ml to avoid contamination with epithelial cells. An additional 5 ml of blood was collected for the analysis of serum tumour markers. The samples for analysis were collected in a tube containing an anticoagulant. The strategy for CTC enrichment was essentially similar to that in the literature [13, 14. Briefly, red blood cell lysis was performed within 12 h of collection of the blood sample. The samples were then resuspended in phosphate‐buffered saline and incubated with magnetic beads coated with anti‐CD45 monoclonal antibody for 30 min, followed by separation of magnetic beads using a magnetic stand. The supernatants were subjected to identification.

### Identification of circulating tumour cells

The identification of enriched CTCs was performed by CD45^−^ fluorescence *in situ* hybridization (FISH), which combined FISH with chromosome 7 and 8 centromere probes and an anti‐CD45 monoclonal antibody (Figure S1 in the Appenidix S2). The CEP 7 and CEP8 probes and specimens were hybridized at 37ºC for 20 min in a hybridizer. Subsequently, the specimens were washed in 50% formamide at 43ºC for 15 min and again immersed in 2× SSC and gradient alcohol. Finally, the specimens were washed twice with 0.2% bovine serum albumin (BSA) and incubated with the CD45 mixture/2% BSA conjugated to Alexa Fluor 594 for 1 h. 4′,6‐Diamidino‐2‐phenylindole (DAPI) was used for nuclear staining for 5 min. Positive CTCs were defined as hyperdiploid CEP8^+^/DAPI^+^/CD45^−^, hyperdiploid CEP7^+^/DAPI^+^/CD45^−^ or hyperdiploid CEP8^+^,CEP7^+^/DAPI^+^/CD45^−^ (Figure S3 in the Appenidix S2). White blood cells were defined as CD45^+^ (Figure S2 in the Appenidix S2) [14].

### Data collection

The clinicopathological data, including patient age and sex, surgical treatment before metastasis (yes or no), palliative surgical treatment (yes or no), lymph node metastasis (yes or no), primary tumour location (left colon or right colon), mutational status, first‐line chemotherapy regimen, targeted therapy, targeted therapeutic drugs, carcinoembryonic antigen (CEA) and carbohydrate antigen 19‐9 (CA199), were collected from patients' records. The CTC data were also collected from the records. Blood samples, including CTC data, CEA and CA199, were obtained within 3 days of initiating treatment or chemotherapy. The CEA, CA199 and CTC levels were tested at three‐cycle intervals at the same time as the baseline evaluation or response evaluation. The CA199 and CEA levels were determined by electrochemiluminescence immunoassay. The normal reference values of CEA and CA199 were 0–5 ng/ml and 0–40 U/ml, respectively. Using the upper limit of normal for CEA (>5 ng/ml) and CA199 (>40 U/ml) as cutoff values, patients were classified into negative or positive groups for each of the markers. Detection of CTCs was performed using epithelial cell adhesion molecule (EpCAM)‐independent enrichment and CD45^−^ FISH. Using the CTC value (≥2) as a cutoff value, based on previous reports [13, 14, cases below or above the cutoff value were classified into negative or positive groups, respectively. A dynamic decrease was defined as two or more consecutive decreases in the levels of the tested markers compared with the last testing during the first‐line treatment. The tumour markers including CEA and CA199 were measured at the Department of Laboratory Medicine of Shanghai General Hospital (Shanghai Jiaotong University School of Medicine, Shanghai, China). The CTCs were measured at the Department of Pathology Shanghai General Hospital (Shanghai Jiaotong University School of Medicine, Shanghai, China).

Survival data were obtained from the medical charts. PFS was defined as the time from the start of chemotherapy to documented disease progression or death, whichever occurred first. OS was defined as the time between the date of diagnosis of mCRC and the date of disease‐related death or last follow‐up visit.

### Assessment of tumour response and follow‐up

Assessment of tumour response was performed every three cycles of treatment by computed tomography or magnetic resonance imaging. The efficacy assessment included complete response (CR), partial response (PR), progressive disease (PD) and stable disease (SD) according to the Response Evaluation Criteria in Solid Tumours version 1.1 (RECIST 1.1) [15]. The best clinical response during chemotherapy for each patient was recorded as the tumour response. Follow‐up was obtained every 3 months after chemotherapy ended. The PFS data, defined as the time from the start of chemotherapy to documented disease progression or death, were obtained from the medical records and telephone follow‐up.

### Statistical analysis

The patient characteristics are expressed as medians (25th–75th percentiles) and categorical data are expressed as numbers (percentages). The relationships between the CTC levels and clinical response were assessed by Fisher's exact test (for categorical variables), Spearman's rank correlation coefficient and Student's *t*‐test (for continuous variables). The correlations between dynamically changing CTCs and the clinical response and tumour markers were also assessed by Fisher's exact test. Survival analysis was performed using the Kaplan–Meier method, and the curves were compared using the log‐rank test. Univariate and multivariate analyses were performed using Cox regression. Statistical analysis was performed using SPSS version 20.0 (SPSS Inc.) software. Statistical significance was set at *p* < 0.05 (two‐sided).

## RESULTS

### Patient characteristics

A total of 79 patients were enrolled in the study (Appendix S1). As shown in Table 1, the median patient age was 63 years. Forty nine (62.0%) patients were men and 30 were women. Most of the patients (49/79) received the FOLFIRI regimen as first‐line chemotherapy, while 17 received the m‐FOLFOX regimen, 8 received the XELOX regimen and 11 received monotherapy (one with S‐1, one with oxaliplatin, one with raltitrexed, three with irinotecan and five with capecitabine) as first‐line chemotherapy. Additionally, 33 patients received biological targeting therapies (20 cetuximab, 13 bevacizumab) at the same time. The median CTC number at baseline of all enrolled patients was 3. Sixty one (77.22%) patients had a CTC number ≥2.

**TABLE 1:** Baseline characteristics of the patients (*N* = 79 colorectal cancer cases)

Characteristic	
Age, median (range) (years)	63 (56–69)
Sex, *n* (%)	
Male	49 (62%)
Female	30 (38%)
Primary tumour location, *n* (%)	
Right colon	18 (22.8%)
Left colon or rectum	61 (77.2%)
Surgical treatment before metastasis, *n* (%)	
No	14 (17.7%)
Yes	65 (82.3%)
Palliative surgical treatment, *n* (%)	
No	52 (65.8%)
Yes	27 (34.2%)
Lymph node metastasis, *n* (%)	
No	38 (48.1%)
Yes	41 (51.9%)
Mutational status, *n* (%)	
No	24 (30.4%)
Yes	55 (69.6%)
First‐line chemotherapy regimen, *n* (%)	
FOLFIRI	43 (54.4%)
FOLFOX or XELOX	25 (31.6%)
Monotherapy	11 (13.9%)
Targeted therapy, *n* (%)	
No	46 (58.2%)
Yes	33 (41.8%)
Targeted therapeutic drugs, *n* (%)	
Cetuximab	20 (25.3%)
Bevacizumab	13 (16.5%)
CEA, median (range) (ng/ml)	38 (9–184.5)
CA199, median (range) (ng/ml)	54 (11–258)
CTCs, median (range)	3 (2–5)

Note

The clinicopathological characteristics are expressed as medians (25th–75th percentiles) and categorical data are expressed as *n* (%).

Abbreviations: CA199, carbohydrate antigen 19‐9; CEA, carcinoembryonic antigen; CTC, circulating tumour cell.

### Correlation between CTC number and the efficacy of systemic chemotherapy

**FIGURE 1:**
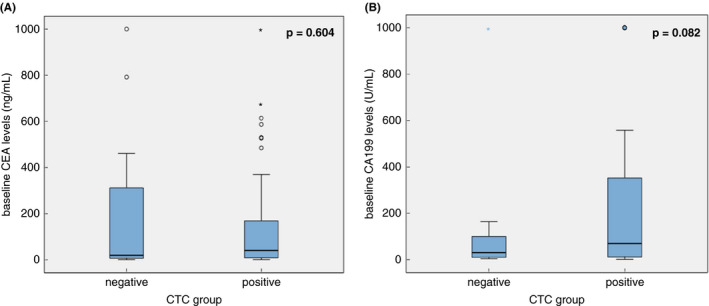
Correlations between the baseline tumour marker levels in different circulating tumour cell (CTC) groups in patients with metastatic colorectal cancer (mCRC). (A) Correlation between the baseline carcinoembryonic antigen (CEA) levels in different CTC subgroups in mCRC patients. (B) Correlation between the baseline CA199 levels in different CTC subgroups in mCRC patients. The *p*‐values were calculated using Student's *t*‐test

Seventy eight patients enrolled in the study were evaluated; one was excluded because of loss to follow‐up. Among the evaluable patients, in the CTC‐positive group 14 had a PR, 27 had SD and 20 had PD and in the CTC‐negative group 3 patients had a PR, 13 had SD and 1 had PD. The baseline CEA and CA199 levels showed no difference between the CTC‐positive and CTC‐negative groups (Figure 1). The baseline CTC levels were significantly associated with the clinical response (*r* = 4.891, *p* = 0.031). CTC number was positively associated with disease progression (*r* = 0.250, *p* = 0.027). No associations were found in this study between the baseline tumour markers (CEA and CA199) and clinical response (Table 2).

**TABLE 2:** Correlation between the baseline tumour markers and clinical response in patients with metastatic colorectal cancer

	*n*	Clinical response			Chi‐square	*p* ^a^	Spearman correlation	*p* ^b^
		PD	SD	PR				
CTCs								
Positive	61	20	27	14	4.891	**0.031**	−0.250	**0.027**
Negative	17	1	13	3				
CA199								
Positive	45	15	19	11	2.221	0.197		
Negative	33	6	21	6				
CEA								
Positive	63	18	32	13	0.452	0.747		
Negative	15	3	8	4				

Bold values are indicate statistical significance.

Abbreviations: CA199, carbohydrate antigen 19‐9; CEA, carcinoembryonic antigen; CTC, circulating tumour cells; PD, progressive disease; PR, partial response; SD, stable disease.

^a^ *p*‐value calculated using the chi‐square test. Statistical significance was set at *p* < 0.05 (two‐sided).

^b^ *p*‐value calculated using Spearman's rank correlation coefficient. Statistical significance was set at *p* < 0.05 (two‐sided).

### Correlation between dynamically decreasing CTCs and the efficacy of systemic chemotherapy

Continuously detected CTCs of 59 patients were available during chemotherapy. The correlation between the dynamic decrease in CTC number and the clinical response was explored. Dynamically decreasing CTC number was significantly correlated with clinical response (*r* = 6.16, *p* = 0.046) (Table 3).

**TABLE 3:** Correlation between the dynamic changing circulating tumour cell (CTC) number and clinical response in patients with metastatic colorectal cancer

	Without CTC reduction (*n* = 31)	With CTC reduction (*n* = 28)	Chi‐square	*p*‐value^a^
PD	8	3	6.16	0.046
SD	19	14		
PR	4	11		

Abbreviations: PD, progressive disease; PR, partial response; SD, stable disease.

^a^ *p*‐value calculated using chi‐square test. Statistical significance was set at *P* < 0.05 (two‐sided).

### Univariate and multivariate analyses according to the clinicopathological data and CTC levels

**FIGURE 2:**
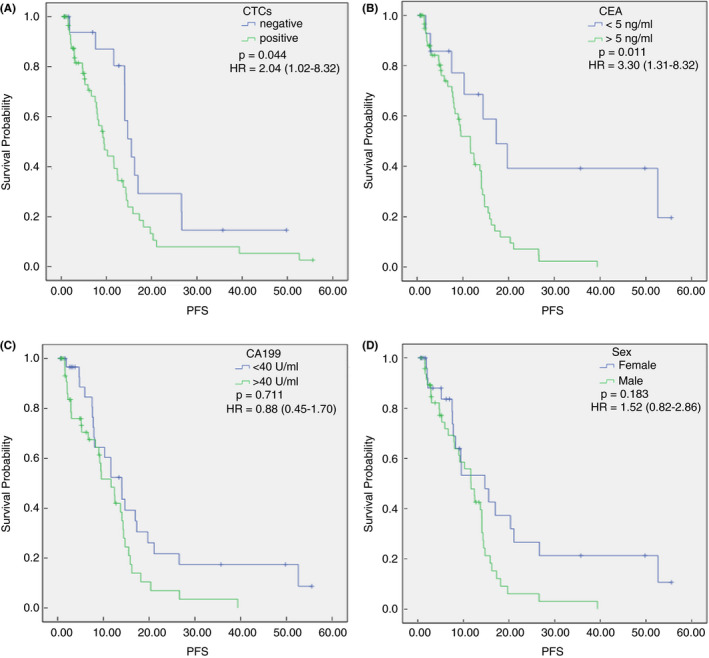
Progression‐free survival (PFS) of patients with metastatic colorectal cancer (mCRC) treated with first‐line chemotherapy. (A) PFS in the circulating tumour cell (CTC)‐positive and CTC‐negative groups. (B) PFS in the carcinoembryonic antigen (CEA)‐high and CTC‐low groups. (C) PFS in the carbohydrate antigen 19‐9 (CA199)‐high and CA199‐low groups. (D) PFS in the different sexes. The PFS was calculated using the Kaplan–Meier method

Univariate analysis showed that male gender [hazard ratio (HR) = 2.01, *p* = 0.025], high CA199 levels (HR = 1.76, *p* = 0.050), high CEA levels (HR = 3.15, *p* = 0.006) and high CTC levels (HR = 1.94, *p* = 0.045) were associated with a poorer PFS. Multivariate analysis revealed that high CEA levels and high CTC levels were poor prognostic factors for PFS (HR = 3.30, *p* = 0.011 and HR = 2.04, *p* = 0.044, respectively; Table 4). The median PFS values were 15.53 and 15.57 months in the CTC‐negative and CEA‐negative groups, respectively, which were significantly higher than those in the positive groups (Figure 2). Thus far, the median OS of these patients has not been reached.

**TABLE 4:** Hazard ratios for the clinicopathological data and inflammatory markers in progression‐free survival

	Univariate analysis		Multivariate analysis	
Characteristics	HR (95% CI)	*p*‐value^b^	HR (95% CI)	*p*‐value^b^
Age (years)^a^				
>63	0.93 (0.60–1.45)	0.777		
≤63	1			
Sex				
Male	2.01 (1.09–3.70)	**0.025**	1.52 (0.82–2.86)	0.183
Female	1		1	
Primary tumour location				
Right colon	1	0.336		
Left colon or rectum	0.73 (0.39–1.36)			
Surgical treatment before metastasis				
Yes	1	0.059		
No	1.84 (0.97–3.47)			
Lymph node metastasis				
Yes	1	0.094		
No	0.62 (0.35–1.08)			
Pathological grade				
High	1	0.824		
Low	0.90 (0.35–2.28)			
Mutational status				
No mutation	0.65 (0.36–1.18)	0.164		
Mutation	1			
Targeted therapy				
Yes	1.03 (0.60–1.77)	0.897		
No	1			
First‐line chemotherapy regimen				
FOLFIRI	0.90 (0.63–1.27)	0.552		
XELOX or FOLFOX				
Monotherapy	1			
CA199 (U/ml)				
>40	1.76 (0.99–3.10)	**0.050**	0.88 (0.45–1.70)	0.711
≤40	1		1	
CEA (ng/ml)				
>5	3.15 (1.39–7.16)	**0.006**	3.30 (1.31–8.32)	**0.011**
≤5	1		1	
CTCs				
≥2	1.94 (1.01–3.73)	**0.045**	2.04 (1.02–8.32)	**0.044**
<2	1		1	

Bold values are indicate statistical significance.

Abbreviations: CA199, carbohydrate antigen 19‐9; CEA, carcinoembryonic antigen; CI, confidence interval; CTC, circulating tumour cell; HR, hazard ratio.

^a^Using the median value as a cutoff value.

^b^Univariate and multivariate analyses were performed using Cox regression.

## DISCUSSION

In this single‐institution retrospective study, we enrolled 78 evaluable patients with mCRC who received first‐line treatment. High baseline CTC levels were associated with a poor treatment response. The dynamic decrease in CTC number was associated with a clinical response. High baseline CTC number and CEA levels were poor prognostic factors for PFS. Compared with the CTC‐positive group, the CTC‐negative group showed a superior PFS in CRC patients receiving first‐line treatment.

CTCs are rare malignant cells found in the bloodstream that originate from the primary tumour or metastatic sites. CTCs may include not only epithelial tumour cells but also tumour cells undergoing epithelial–mesenchymal transition and tumour stem cells [16]. The failure of CTCs to execute the anoikis programme could result in cancer cells surviving in the bloodstream. The mechanisms of resistance of CTCs to anoikis may include changes in the integrin repertoire, activation of oncogenes, overexpression of growth factor receptors and changes in the tumour microenvironment [17]. CTCs are a potential surrogate for distant metastasis and are a novel and promising biomarker for the diagnosis and therapy of various malignancies. The US Food and Drug Administration has approved the use of CTCs in the diagnosis and treatment of patients with mCRC, prostate cancer and breast cancer [18, 19, 20.

Numerous technologies that rely on the physical and biological properties of CTCs have been developed to enrich CTCs from normal haematopoietic cells. These enrichment techniques can broadly be divided into immunocapture methods that differentiate cells based on epithelial cell surface marker expression, notably EpCAM, and those that differentiate on the basis of distinct biophysical properties [21]. The CellSearch system, which is based on biological features, is the gold standard platform for the isolation, enrichment and detection of CTCs [21]. Paramagnetic beads coupled with antibodies against the EpCAM are used for CTC enrichment by this method. Detection of CTCs in addition requires the presence of epithelial cytokeratins and the absence of the leukocyte marker CD45 in nucleated cells [22]. Enumeration of CTCs using the CellSearch method is a useful clinical predictive marker for therapeutic response and survival in patients with mCRC [23, 24. Sastre et al. quantified CTCs in 7.5 ml of blood collected from 97 patients and 30 healthy volunteers using the CellSearch system. The cut‐off of 2 CTCs/7.5 ml was used to define the test as positive. Positive CTCs were detected in 34 of 94 patients (36.2%) [25]. In clinical practice the recovery rates range from 42% to 90% and the clinical detection rate was between 20% and 77.5% [21]. Maria Gazouli et al. also presented an assay incorporating cadmium selenide quantum dots (QDs) to detect CRC CTC surface antigens. The principle of their assay is the separation of CTCs from body fluids using magnetic beads in conjunction with QDs, using an anti‐EpCAM antibody and a monoclonal anticytokeratin 19 antibody. The accuracy of the QD detection system, as evaluated using clinical samples from CRC patients, ranged between 78.57% and 85.71%. [26]. The authors also successfully found that DNA mutational analysis of CTCs may enable noninvasive, specific biomarker diagnostics and expand the scope of personalized medicine for cancer patients [27].

However, EpCAM expression on the surface of epithelial tumour cells is highly heterogeneous, and some types of cancer cells do not express EpCAM. The clinical application of such techniques to isolate CTCs shed from different solid tumours is limited [13]. An EpCAM‐independent enrichment approach is necessary to avoid the loss of epithelial markers. FISH detection with multiple chromosome probes may contribute to the detection of CTCs [28, 29. Chen et al. designed a modified strategy to enrich CTCs using EpCAM‐independent enrichment and to detect CTCs with CD45^−^ FISH that combined double probes of CEP8, ‐7 and an anti‐CD45 antibody for detection to improve the positivity rate. In their study, 84% of patients had a CTC number ≥2 before treatment, and the results showed that the sensitivity of CTCs for diagnosis was 84% and the specificity was 97.6% with dual probes [14]. Li et al. also used combined negative enrichment, immunocytochemistry, CD45 staining and FISH to identify, enumerate and characterize CTCs. The results showed the multiploid cell rates of four cancer cell lines were >96%. Using a cutoff value of 2 CTCs, the positive rate of detecting lung, gastric, breast and oesophageal cancers was 71.33%, 86.21%, 76.77% and 78.35%, respectively [30]. Similar to previous studies, we used a combined negative enrichment method, immunocytochemical CD45 staining and FISH, which combined double probes of CEP8 and ‐7 and an anti‐CD45 antibody, for detection of CTCs to investigate the impact of CTC number on the outcomes and prognoses related to first‐line treatment in mCRC patients. A CTC value (≥2) was used as a cutoff value because of the efficacy reported in previous studies [14, 25, 30.

In our study, CTC number was significantly correlated with the clinical response in patients with mCRC who received first‐line treatment. Patients in a CTC‐negative group and with CTC reduction during chemotherapy revealed a good prognosis, consistent with previous studies [18, 19, 20, 24. Aggarwal et al. analysed the relationship between CTC number and CEA and OS in patients with mCRC. The patients with a low baseline CTC number had a longer survival, and the baseline CTC number independently predicted survival [10]. Cohen et al. enrolled 430 mCRC patients in a prospective multicentre study and found that CTC number before treatment was an independent predictor of PFS in patients with mCRC. Patients with an unfavourable baseline CTC number were associated with an inferior median PFS [21, 22. Shen et al. reported that the baseline CTC count could be a valuable predictive and prognostic biomarker for patients with small cell lung cancer who received first‐line chemotherapy. The reduction of CTC number after two cycles of chemotherapy was a potential predictor of chemotherapeutic response in small cell lung cancer [24].

High baseline CEA levels were prognostic factors for PFS in the univariate and multivariate analyses. However, no associations were found between the baseline tumour markers and clinical response in Fisher's exact test, which may be related to the length of follow‐up. Some enrolled patients accepted the first‐line therapy and did not reach the PFS time. Additional samples and a longer follow‐up time are required to validate the efficacy of this tumour marker in the clinic.

Our study had some limitations. The small sample size included from a single medical centre might have limited the statistical significance of the impact of the clinical variables on the survival outcome. Additionally, the different first‐line chemotherapy regimens in the enrolled patients might have been confounded by CTCs relevant to PFS, the tumour response and prognostic analysis. Furthermore, the short follow‐up time might have limited the conclusions that could be drawn. The present results require more samples for further validation.

## CONCLUSION

Positivity for CTCs at baseline without a reduction in CTC number during chemotherapy in mCRC patients was positively correlated with PD. Higher CTC numbers are related to a poor prognosis. Because blood collection is simple, convenient and minimally invasive, CTC number may be used as a new biomarker for predicting disease progression and survival. The relationship between CTC number and efficacy of chemotherapy in patients with mCRC should be investigated further.

## ETHICS STATEMENT

The study was approved by the Ethics Board of Shanghai General Hospital.

## PATIENT CONSENT STATEMENT

Informed consent to participate in the study was obtained from all the patients.

## PERMISSION TO REPRODUCE MATERIAL FROM OTHER SOURCES

No material has been reproduced from other sources.

## CONFLICT OF INTEREST

The authors declare that they have no conflicts of interest.

## AUTHOR CONTRIBUTIONS

Li Qi conceived of the presented idea. All authors collected the data. Shen Feifei and Zhu Yiwen analysed, interpreted the data and wrote the manuscript. Li Qi and Liu Chuan supervised and supported the study and critically revised the manuscript. All authors approved the final version of the manuscript.

## Supporting information

Appendix S1

Appendix S2

## Data Availability

The data that support the findings of this study are available from the corresponding author upon reasonable request.
