# Supplementary material for: Clinical significance of circulating tumour cells and tumour marker detection in the chemotherapeutic evaluation of advanced colorectal cancer
Source: Colorectal Dis. 2021 Oct 23;24(1):68–76. doi: 10.1111/codi.15939 (PMC9298334; doi:10.1111/codi.15939)
Supplement: Supplementary file 2 — Appendix S2 [file CODI-24-68-s002.docx]

**Supplementary Information**

**Figure S1. Identification of CTCs.** A. CD45^-^ and CD45 are specific antigens on the surface of white blood cells, and CD45^-^ is used to exclude white blood cells. B. DAPI^+^ and DAPI bind firmly to DNA, and tumor cells are DAPI^+^. C. CEP7^+^ and chromosomes of tumor cells are amplified. The normal number is 2 in CEP7, while 3 chromosomes in CEP7 identified as abnormal cells; D. CEP8^-^, the normal number is 2 in CEP8. E. synthesis, synthetic images of figure A to D.

**Figure S2. Identification of white blood cells.** A. CD45^+^, white blood cells were defined as CD45pos; B. synthesis, synthetic images.

**Figure S3. Positive CTCs.** A) The results of the FISH assay showed that there were 2 CEP8 triploids and 1 CEP7 polyploid (WBC was the control, and the peripheral red circle suggested CD45 staining). B) The results of FISH detection showed that there were 5 triploids of CEP8 and 1 triploid of CEP7 (WBC was the control, and the peripheral red circle suggested CD45 staining).


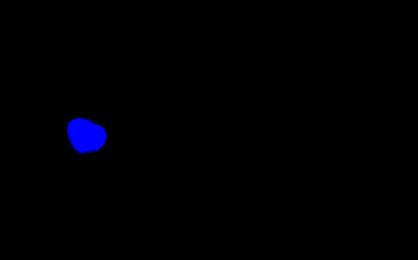

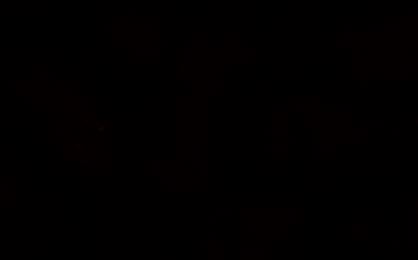


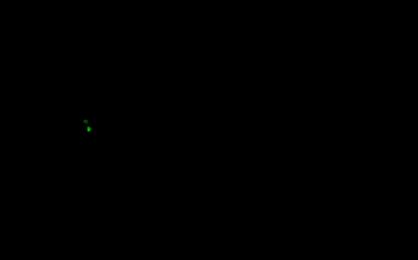

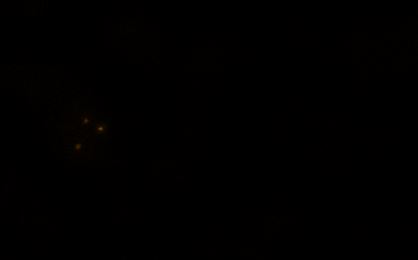
 A: CD45^-^ B: DAPI^+^

C: CEP7^+^ D: CEP8^-^


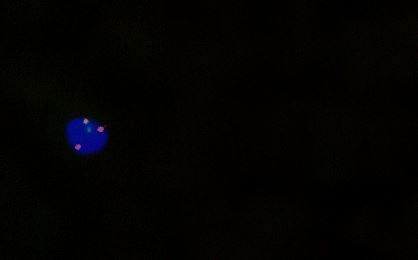


E: Synthesis

**Figure** **S1. Identification of CTCs.**


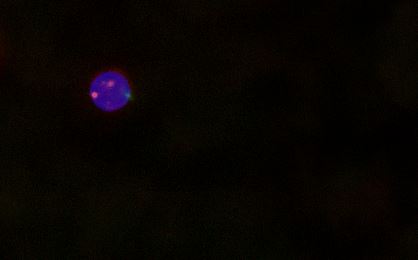

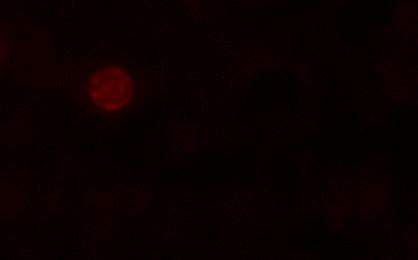


A: CD45+ B: Synthesis

**Figure S2. Identification of white blood cells.**

aA
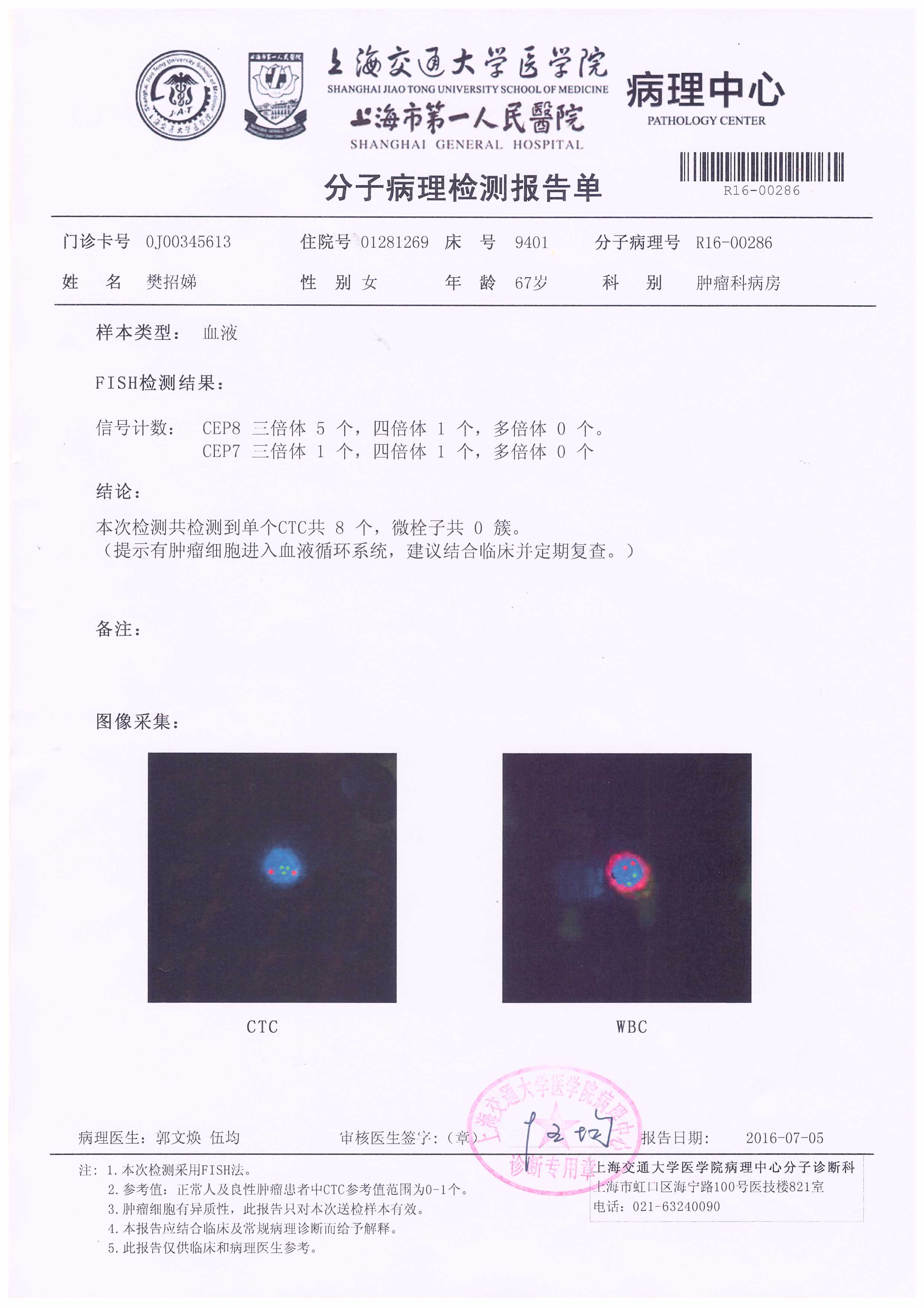


A CTC WBC


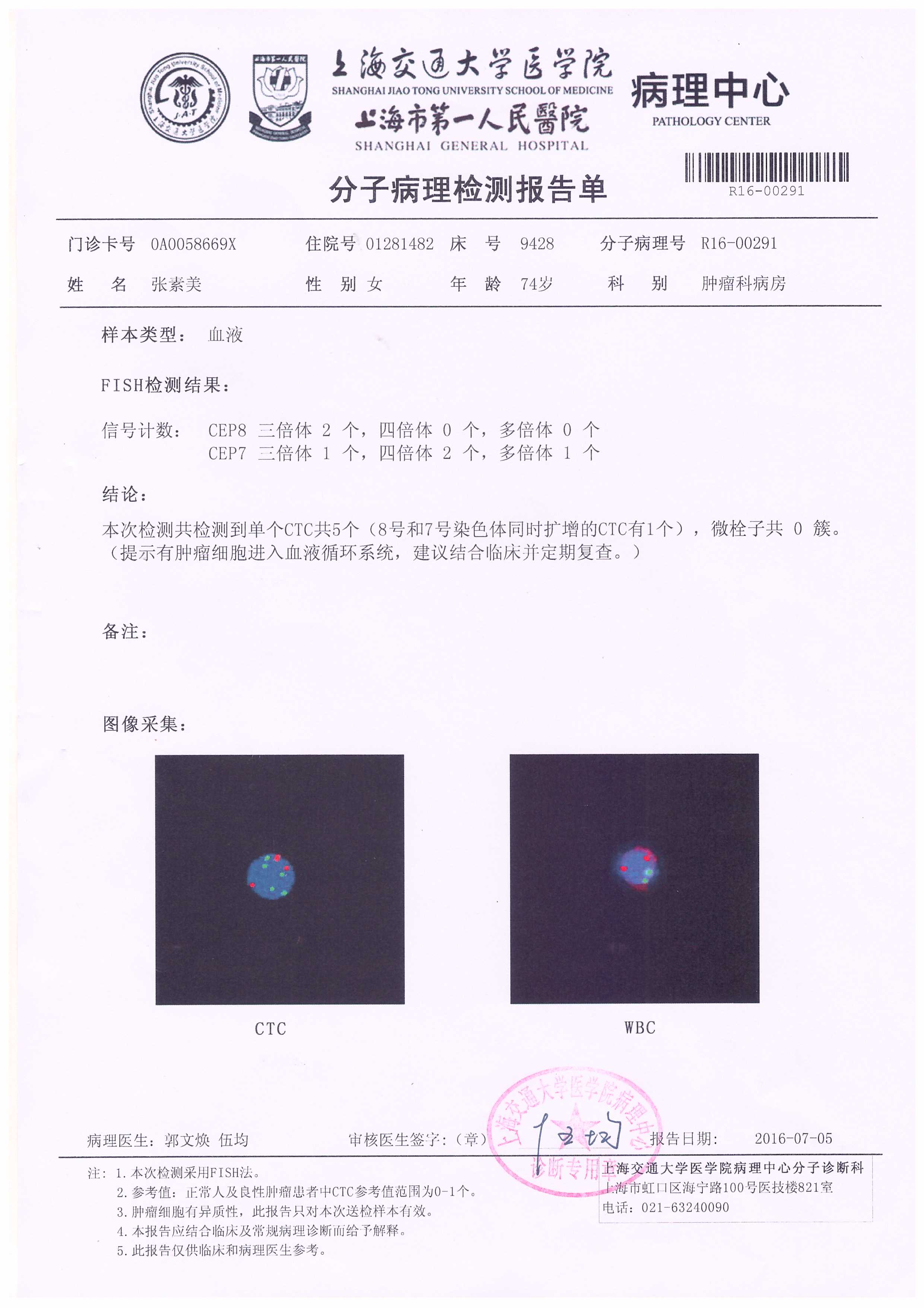


B CTC WBC

**Figure S3. Positive CTCs.**
